# A Sparse Representation-Based Algorithm for Pattern Localization in Brain Imaging Data Analysis

**DOI:** 10.1371/journal.pone.0050332

**Published:** 2012-12-05

**Authors:** Yuanqing Li, Jinyi Long, Lin He, Haidong Lu, Zhenghui Gu, Pei Sun

**Affiliations:** 1 Center for Brain Computer Interfaces and Brain Information Processing, South China University of Technology, Guangzhou, People's Republic of China; 2 Institute of Neuroscience, State Key Laboratory of Neuroscience, Shanghai Institutes for Biological Sciences, Chinese Academy of Sciences, Shanghai, People's Republic of China; 3 Laboratory for Cognitive Brain Mapping, RIKEN Brain Science Institute 2-1 Hirosawa, Wako, Saitama, Japan; 4 Department of Psychology, Tsinghua University, Beijing, People's Republic of China; University of Minnesota, United States of America

## Abstract

Considering the two-class classification problem in brain imaging data analysis, we propose a sparse representation-based multi-variate pattern analysis (MVPA) algorithm to localize brain activation patterns corresponding to different stimulus classes/brain states respectively. Feature selection can be modeled as a sparse representation (or sparse regression) problem. Such technique has been successfully applied to voxel selection in fMRI data analysis. However, single selection based on sparse representation or other methods is prone to obtain a subset of the most informative features rather than all. Herein, our proposed algorithm recursively eliminates informative features selected by a sparse regression method until the decoding accuracy based on the remaining features drops to a threshold close to chance level. In this way, the resultant feature set including all the identified features is expected to involve all the informative features for discrimination. According to the signs of the sparse regression weights, these selected features are separated into two sets corresponding to two stimulus classes/brain states. Next, in order to remove irrelevant/noisy features in the two selected feature sets, we perform a nonparametric permutation test at the individual subject level or the group level. In data analysis, we verified our algorithm with a toy data set and an intrinsic signal optical imaging data set. The results show that our algorithm has accurately localized two class-related patterns. As an application example, we used our algorithm on a functional magnetic resonance imaging (fMRI) data set. Two sets of informative voxels, corresponding to two semantic categories (i.e., “old people” and “young people”), respectively, are obtained in the human brain.

## Introduction

One fundamental question in neuroscience focuses on determining how information is processed within local and global networks in the brain. Recently, multivariate pattern analysis (MVPA) approaches have been used successfully in revealing brain patterns activated by different stimulus conditions in brain imaging studies [1], [2], [3], [4], [5], [6]. Three common strategies have been employed to determine where the brain contains discriminative information for different stimulus categories, and this is known as a pattern localization procedure. Based on prior knowledge, multivariate analysis can be restricted to anatomically or functionally predefined brain regions [7], [8], [9], [10]. An alternative method is a local multivariate search approach (e.g. the searchlight algorithm), in which features are evaluated in local brain regions first and then all of these local features are combined to form a whole-brain information mapping [11], [12]. The third strategy is a whole-brain approach which treats all features/voxels as a vector and does not need a priori information on the location of the informative features. Whole-brain approach-based feature selection algorithms have been used to reveal fine-grained spatial discriminative patterns both in simulations and real functional magnetic resonance imaging (fMRI) data analysis [1], [5], [13], [14].

Ideally, all of the informative features contributing to the discrimination should be extracted, no matter how small a contribution they provide. However, in most MVPA algorithms, not all the informative variables are selected because part of the informative variables may be sufficient for decoding or classification. However, when trying to extract the sufficient informative features, those representing noise may be selected; therefore follow-up statistical tests are necessary.

For the purpose of identifying all the informative features, we proposed a sparse representation-based pattern localization algorithm combined with a nonparametric statistical test in this study. We summarized the process of the algorithm as three components: a K-fold cross-validation of recursive feature search where feature weights were determined by a sparse representation method, construction of two probability maps based on the selected features, and a permutation test at the individual or group level. In our previous study [15], we established a sparse representation-based multivariate algorithm for voxel selection in fMRI data analysis. Furthermore, our data analysis results demonstrated its better performance in detecting subtle difference between two different brain states than several conventional univariate methods e.g. the generalized linear model (GLM) method [3], [16], [17], [18]. Compared with the methods in [15] and other related studies, the contributions of the algorithm in this paper were three folds: 1) During the recursive feature search, the informative features selected by sparse representation were eliminated recursively until the decoding accuracy dropped to a threshold close to chance level. In this way, most of the informative features were expected to be identified/selected; 2) The positive and negative signs of the feature weights obtained by sparse representation were associated with the two stimulus conditions/brain states respectively. Hence the selected features were separated into two sets according to the signs of the weights; 3) The permutation test guaranteed the rejection of the irrelevant/noisy features in the above two selected feature sets. Thus, two patterns corresponding to two stimulus classes/brain states respectively were localized. We demonstrated the effectiveness of our approach using a toy data set and an intrinsic signal optical imaging data set. Furthermore, we illustrated the application of our approach using an fMRI data set.

## Materials and Methods

### 1 Preliminary: feature selection modeled as sparse representation (or sparse regression) problem

The neuroimaging data were given by a matrix 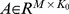, where the 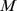 rows and the 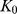 columns corresponded to the time points and the features/variables (e.g. voxels in the fMRI data or pixels in the optical imaging data), respectively. The column vector 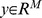 was a function with label information to be regressed. For instance, 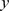 could be a stimulus function in an fMRI experiment with 1 representing stimulus and 0 representing no stimulus, or a label vector with 1 representing the first class and −1 representing the second class.

Feature selection was based on the weights of all features, which were determined by sparse representation [15]. We outline the algorithm for weight determination below.

### Sparse representation method for feature/variable weight determination

For a data matrix 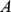 and a column vector 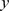, we solved the following optimization problem to obtain a weight vector 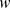 of variables.
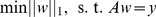
(1)


Model (1) can be seen as a sparse regression between the data matrix 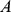 and the function 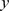 with label information. The optimal solution of (1) is denoted by 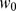. The absolute value of each entry of 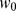 reflects the contribution of its corresponding variable to the regression between the data matrix 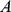 and the function 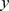 or to the discrimination between two classes when 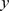 is constructed using the labels 1 and −1.

The optimization problem (1) can be converted to a standard linear programming problem as below [15].

Setting 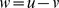, where 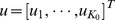 and 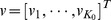 are nonnegative, model (1) can be converted to the following linear programming problem with nonnegative constraints,
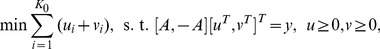
(2)which can be solved using the Matlab optimization toolbox.

Here we could also use another regression approach, e.g., support vector machine (SVM), instead of sparse representation for weight determination. A difference between the two methods is: the weights obtained by sparse representation is sparse while those obtained by SVM are dense. Sparse weights are useful for highlighting those variables relevant with the labels or the vector to be regressed [15]. Additionally, the signs of weights obtained by sparse representation are related to the classes of data, as demonstrated by data analysis and mathematically proven based on several simplified models in this paper. This characteristic of SVM has also been demonstrated by simulations in this paper and proposed in several other studies [2], [14]. We mainly used sparse representation for determining weights of features/variables in this study.

### 2 Proposed sparse representation-based pattern localization (SPL) algorithm

**Figure 1:**
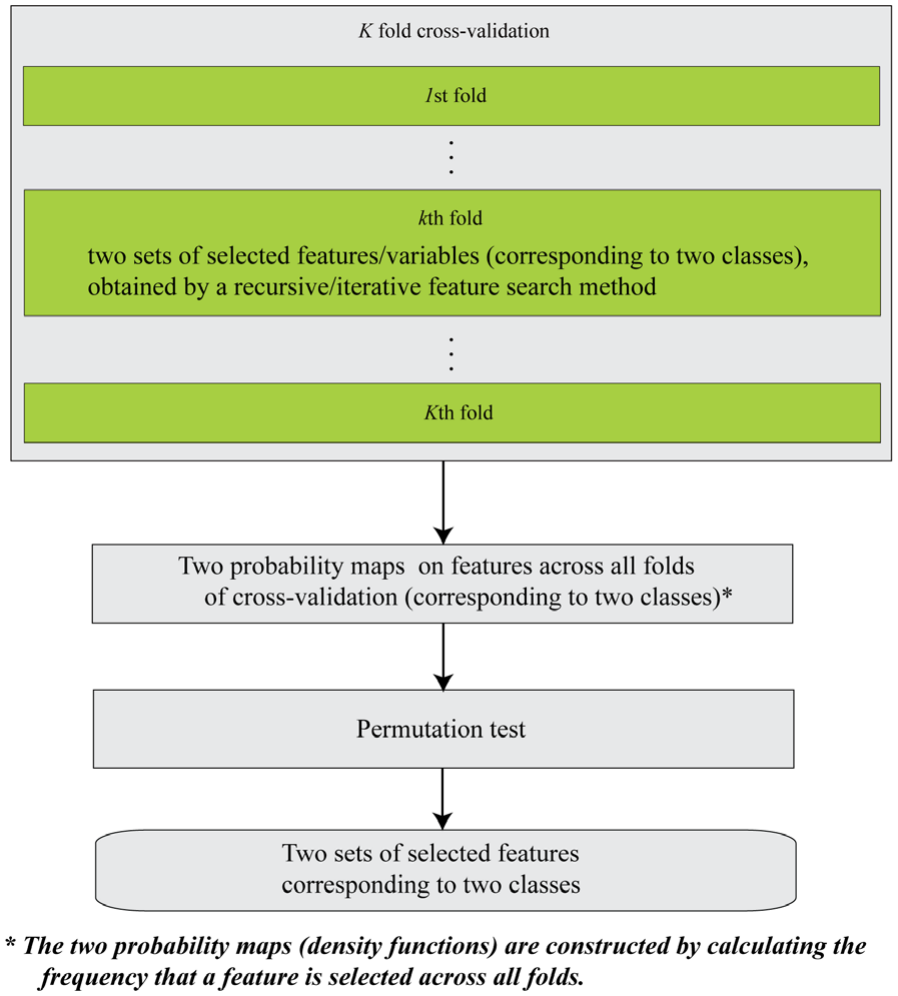
SPL algorithm diagram.

We first present the outline of our SPL algorithm here. As shown in Figure 1, a 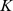-fold cross-validation was performed with the SPL algorithm. In each fold, a recursive iterative feature elimination method relying on the weights obtained by sparse representation was used to pick up as many informative variables as possible, and these selected features were divided into two sets according to the signs of their weights corresponding to two stimulus classes/brain states. Next, two probability maps/density functions were constructed using the two classes of features selected across all the 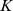 folds of cross-validation. Inside each probability map, the probability value of a feature was obtained by counting the number of times the feature was picked up across all folds. To remove the irrelevant features, these two probability maps were tested with a permutation test at the individual level or tested at the group level if a group of data were available. Thereafter, two patterns corresponding to two stimulus classes/brain states were obtained. In the following, we explain the SPL algorithm step by step.

#### 2.1 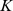-fold cross-validation of recursive feature search

Regarding the cross-validation, both the data matrix 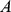 and the column vector 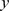 were equally partitioned into 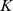 non-overlapping parts according to their rows. In the 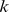-th fold (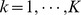), the 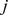-th part was removed and the rest (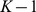) parts (denoted as 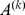 and 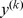 in the following) were used for searching two sets of informative features, which correspond to two classes of stimuli/brain states. This was implemented by a procedure of sparse representation and recursive feature search, as described in the following. Regarding parameter 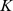, the number of folds, we suggest that it is set larger than 20 since it is related to the calculation of probability maps as shown later.

### Recursive feature search in each fold of cross-validation

In each fold of cross-validation, we recursively eliminated informative features selected by the sparse regression method until the decoding accuracy based on the remaining features drops to a threshold close to the chance level 50% for the two-class problem. According to the signs of the sparse regression weights, these identified features were separated into two sets corresponding to two stimulus classes/brain states respectively. In this way, the resultant two feature sets including all the identified features were expected to involve as many informative features for discrimination as possible.

**Figure 2:**
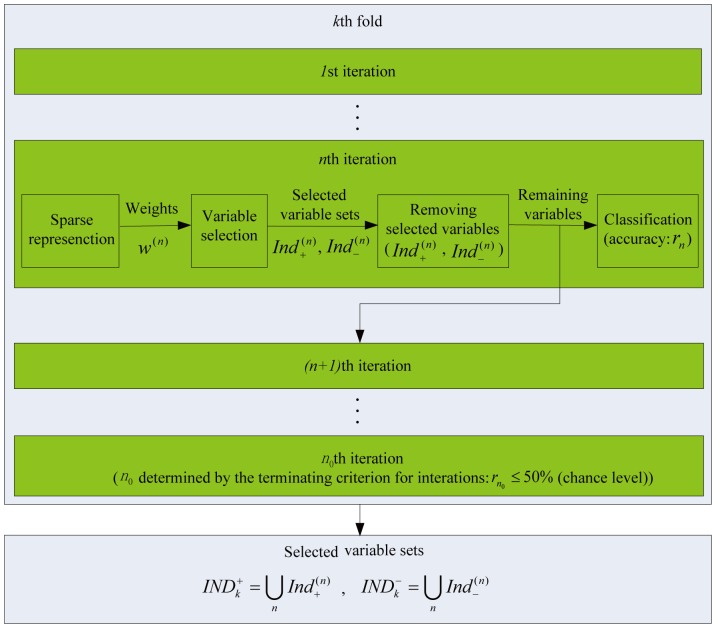
The algorithm diagram for the recursive feature search in a fold of cross-validation.

The process of the recursive feature search in the 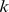
th fold is illustrated in Figure 2. In the first iteration, the data were the matrix 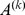 and column vector 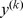. In the 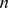
th iteration (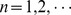), we performed the following four steps:

Step 1: Sparse representation for feature weight determination. We applied the sparse representation method to the data updated in the previous iteration and obtained a weight vector denoted as 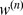 of variables.

Step 2: Feature selection. We used the weight vector 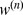 to determine two sets denoted as 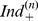 and 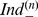, each containing 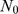 informative variables/features. The two sets 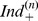 and 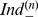 corresponded to the 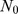 largest positive elements and the 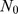 smallest negative elements of the weight vector 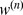, respectively.

Step 3: Informative feature removal. We removed these variables in 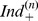 and 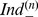 from the data matrix used in the current iteration, and the updated data matrix composed by the remaining variables was used in the next iteration.

Step 4: Decoding. We performed a decoding by applying an SVM classifier to the updated data matrix, and the prediction accuracy of the labels, denoted as 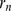, was calculated based on a cross-validation classification procedure (e.g. 20 folds of cross-validation for classification in this paper). The cross-validation here was based on the training data updated in the 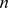
th iteration, which was separated into the training and test data sets for classification. Specifically, this cross-validation was only for calculating the prediction accuracy 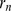 and subordinate to the higher level one described above.

Terminating criterion: The above iteration procedure ran until the prediction accuracy dropped to a threshold. Theoretically, the best threshold was the chance level 50% for the two-class problem, which was used in this paper.

After the iterations in the 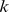
th fold terminated, the two selected variable sets corresponding to the two stimulus conditions respectively were
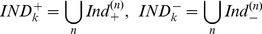



Remark 1: (i) In the above Step 2, we assumed that the signs of those weights obtained by sparse representation were associated with the classes of stimuli/brain states. In Appendix S1, we presented the mathematical proof based on several simplified models to illustrate the rationality of this assumption. (ii) In this recursive feature search method, there was a parameter 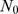 (the number of variables with the largest positive/the smallest negative weights selected in each iteration). In sparse representation, the number of nonzeros in the optimal weight vector is generally equal to the number of equations (Li, et al., 2004). Thus the parameter 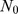 should be less than the half of the number of equations in the optimization problem in each iteration. If we set a smaller 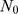, then more iterations are needed to extract all informative variables. We can set the parameter 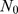 according to the total number of features of data. For instance, we could set 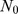 to about 50 for fMRI data according to our experience. (iii) In the above algorithm, the test data of the 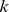
th fold were not used. Through removing the test data set in each fold of the cross-validation, different training data sets were obtained. The features representing noise, which were selected based on different training data sets, were generally different. On the contrary, the features, which were frequently selected in the cross-validation, were potentially informative.

After 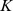 folds of cross-validation, we obtained 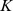 sets 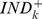 of selected features corresponding to a stimulus class or brain state, and 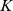 sets 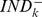 of selected features corresponding to another stimulus class or brain state. The two classes of selected features were used to construct two probability maps (density functions) respectively, as described in the following subsection.

#### 2.2 Two probability maps for pattern localization

As described above, two sets of selected variables, which correspond to two classes of stimuli/brain states respectively, were obtained in each fold of cross-validation. Using the 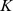 sets 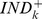 of selected features corresponding to a stimulus condition or a brain state, we constructed a probability map. The probability value of each feature measures the preference of this feature to this stimulus condition or brain state. It was calculated by counting the number of appearances of this feature in all of the 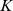 sets 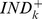 and dividing this number by the total number of selected features in those sets. Similarly, using the 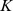 sets 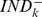 of selected features corresponding to the other stimulus condition or brain state, another probability map was constructed. The two probability maps will be used for permutation test as described in the following.

#### 2.3 Permutation tests at the individual subject level and group level

In each fold of the cross-validation, the two feature sets obtained by the recursive feature search included as many informative features for discrimination as possible. However, some irrelevant/noisy features were unavoidably selected by the method. To determine if a feature had contribution to the discrimination between two classes of stimuli/brain states, we performed a permutation test at the individual subject level or the group level on each of the two probability maps, described below.

Permutation test at the individual level: In each permutation, we randomly gave the labels for the data and repeated the above procedure of cross-validation, and obtained two probability maps/density functions. A null distribution for each class was constructed by pooling all probability values of the 100 probability maps corresponding to this class, which were obtained through the 100 permutations [19]. Next, for a given significance level 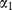, we found two critical thresholds 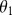 and 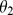 corresponding to the percentile 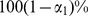 of the two null distributions. Finally, we applied the two thresholds 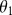 and 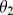 to the two probability maps respectively and obtained two sets of selected features. For a multiple comparison correction, we may use a strict significance level e.g. 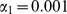 or a cluster size e.g. 10 in fMRI data analysis.

Permutation test at the group level: For each subject, we obtained two probability maps with respect to features corresponding to two classes respectively as in Sections 2.2.1 and 2.2.2. We averaged these probability maps across all subjects and obtained two average probability maps also corresponding to two classes respectively. Next, we performed multiple permutations (e.g. 100). In each permutation, we randomly gave labels for the data from each subject and obtained two probability maps for each subject, and further calculated two average probability maps across all subjects. A null distribution for each class was constructed by pooling all probability values of the 100 average probability maps corresponding to this class, which were obtained through the 100 permutations. For a given significance level 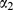, we found two critical thresholds 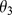 and 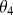 corresponding to the percentile 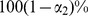 of the two null distributions respectively. Finally, using the two thresholds and their corresponding probability maps, we obtained two sets of selected features, corresponding to two classes. For a multiple comparison correction, we may use a strict significance level e.g. 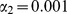 or a cluster size e.g. 10 in fMRI data analysis.

### 3. Experimental design and data acquisition

#### Experiment 1: Simulations

There were two simulations in this experiment. In the first simulation, we showed that when the data were sufficient, i.e., the number of known data vectors was much larger than that of the variables, two patterns (two sets of informative variables) corresponding to two classes of data could be well estimated by directly using the weights with signs determined by sparse representation or SVM. We also demonstrated that the signs of these weights were associated with the two classes of the data. In the second simulation, we considered the case where the number of known data vectors was much smaller than that of the variables. We illustrated our approach for localizing two patterns and demonstrated its effectiveness. We also used SVM to replace sparse representation in our SPL algorithm for weight determination and obtained comparable results.

In the first simulation, we considered the following optimization problem, which is a sparse representation model similar to equation (1),
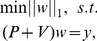
(3)where 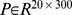 is a pattern matrix, of which each of the first 10 rows was the pattern 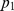 and each of the last 10 rows was the pattern 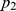. The two fixed pattern vectors 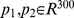, the first 25 entries of 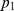 and the last 25 entries of 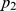 took value 1, and the other entries were zero. 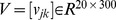 was a noise matrix, of which each column was from colored Gaussian noise with zero mean and variance 1. The average temporal SNR was −7.5 dB, whereas the average spatial SNR was −17 dB. Furthermore, 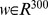 was an unknown weight vector with 300 variables, which was determined by solving the optimization problem, and 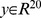 was a label vector with the first 10 entries being 1 and the last 10 entries being −1. Thus the first 10 constraint equations of (3) represented the first class, while the last 10 represented the second class.

Remark 2: In this paper, the temporal SNR for a column of the data matrix 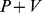 was calculated as: 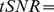 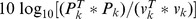, where 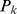 was a nonzero column of the pattern matrix 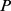, and 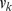 was a column of the noise matrix 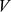. The average tSNR was obtained by averaging tSNR across all nonzero columns of the pattern matrix 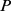. The spatial SNR for a row of the data matrix 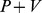 was calculated as: 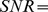 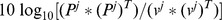, where 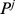 was a row of the pattern matrix 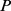, and 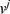 was a row of the noise matrix 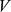. The average SNR was obtained by averaging SNR across all rows of the matrix 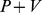.

Note that in (3), the data matrix 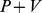 and the label vector 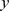 were known, but the two patterns 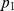 and 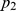, and the noise matrix 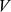 were unknown. We solved the optimization problem (3) for 500 times to find the two pattern vectors 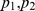. Each time, only the noise matrix was regenerated, whereas the two pattern vectors were unchanged. We calculated the average weight vector across the 500 repeats and localized the two patterns 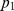 and 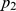 using the average weight vector. For the purpose of comparison, we also used SVM instead of the sparse representation model (3) for determining the weights and searching the patterns 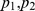. Note that the 500 repeats of solving the optimization problem (3) in this simulation implied that we had 10,000 known data vectors with labels (each data vector was a row of the matrix 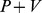). However, in a real-world experiment, it is difficult to collect such a large data set.

In the second simulation, we used a much smaller data set to find two patterns using our approach. Considering the model (3), we first we generated two pattern vectors 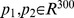 each containing 25 nonzeros with their positions randomly assigned and 275 zeros and thus obtained the pattern matrix 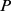. Each nonzero entry of 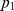 and 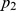 took value 1. Furthermore, the index sets of nonzeros of the two patterns were non-overlapped. We then generated the noise matrix 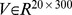 as in the first simulation, where the average temporal SNR and the average spatial SNR were −7.6 dB and −17.1 dB respectively. The 20 rows of 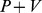 were separated into two classes, the first containing the pattern 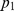 were labeled as 1, and the second containing the pattern 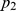 were labeled as −1. For better localizing the two patterns 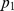 and 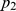, we regenerated the data 5 times as above (corresponding to 5 subjects in a real-time experiment, e.g., an fMRI experiment). Each time, only the noise matrix were regenerated, whereas the two patterns 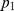 and 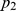 were fixed. We performed our SPL algorithm with a permutation test at the group level, and predicted the two patterns 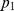 and 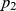. We also used a linear SVM to replace sparse representation method in our SPL algorithm for determining the weights and searched the two patterns 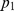 and 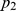. Note that in this simulation, we used 100 known data vectors with labels to localize the two 300 dimensional patterns (each data vector was a row of the matrix 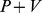), and the number of known data vectors was much smaller than that of variables.

For comparison, we used a standard SVM and the univariate correlation method for localizing the two patterns 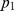 and 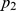. We applied a SVM to the above data set containing 100 known data vectors and obtained a weight vector. For each variable, we also calculated the correlation coefficient between the 100 dimensional data vector corresponding to this variable and the label vector. Using the weight vector or these correlation coefficients, we localized the two patterns 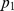 and 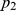.

#### Experiment 2: Optical imaging data acquisition

In this experiment, intrinsic signal optical imaging [20] was used to collect data from the primary visual cortex of a Macaque monkey. The frame size was 100×100 pixels, covering an approximately 4×4 mm cortical area. Images were acquired at a frame rate of 4 Hz. Each trial lasted 4 seconds, and the stimulation started at 0.5 s and ended at 4 s. Baseline fluctuation in each trial was reduced. Mean responses of between 1.75 and 3 s were used in the data analysis because the response reached its maximum during this period. There were four stimulus conditions, i.e., random dots drifting toward the right (0 degree, c1), upward (90 degree, c2), to the left (180 degree, c3) and downward (270 degree, c4). In each condition, 40 trials were carried out. In this study, only 40 horizontal axis-of-motion trials (sum of each 40 trials from c2 and c4) and vertical axis-of-motion trials (sum of each 40 trials c1 and c3) were used. The detailed experimental procedures have been described elsewhere [21].

#### Experiment 3: fMRI data acquisition

In this experiment, fMRI data were acquired from nine human subjects. This study was approved by the Ethics Committee of Guangdong General Hospital, China, and all subjects gave their written informed consent for the study. The visual stimuli were 80 grayscale pictures of Chinese faces at two different age levels (40 old persons and 40 young persons). During each trial, which lasted 10 seconds or 5 volumes (TR = 2 s), the subject was instructed to make a covert semantic categorization (old vs. young) based on the pictures. Each picture was used only once in one trial, and 80 trials were collected for each subject. Mean responses of third, fourth and fifth volumes in each trial were used, whereas the other volumns were discarded because of the delay of BOLD response.

Preprocessing consisted of head motion correction, slice timing correction, coregistration between functional scans and structural scan, normalization to a MINI standard brain, data masking to exclude nonbrain voxels, time series detrending and normalization of time series to a zero mean and a unit variance.

To reduce the amount of computation and remove noise, initial voxel selection was performed using a correlation method. Cross-correlation was performed voxel by voxel between BOLD responses and stimulus function, the top 2,500 voxels with correlation coefficient values larger than 0.15 were selected for later processing.

## Results

We demonstrated the effectiveness of our analysis method and illustrated its application through three experiments. The first experiment contained two simulations based on toy data. The second was an orientation preference experiment in the monkey visual cortex using an intrinsic signal optical imaging technique. The third was an fMRI experiment for face recognition in the human brain.

### Experiment 1: Simulations

There were two simulations conducted in this experiment. In the first simulation, we showed that when the data were sufficient, two patterns could be estimated by directly using the weights with signs determined by sparse representation or SVM, and that the signs of these weights were associated with two classes. In the second simulation, we illustrated our approach and showed that the two patterns could be localized when the data were insufficient. We also used a linear SVM instead of sparse representation for weight determination in our SPL algorithm and obtained comparable results.

**Figure 3:**
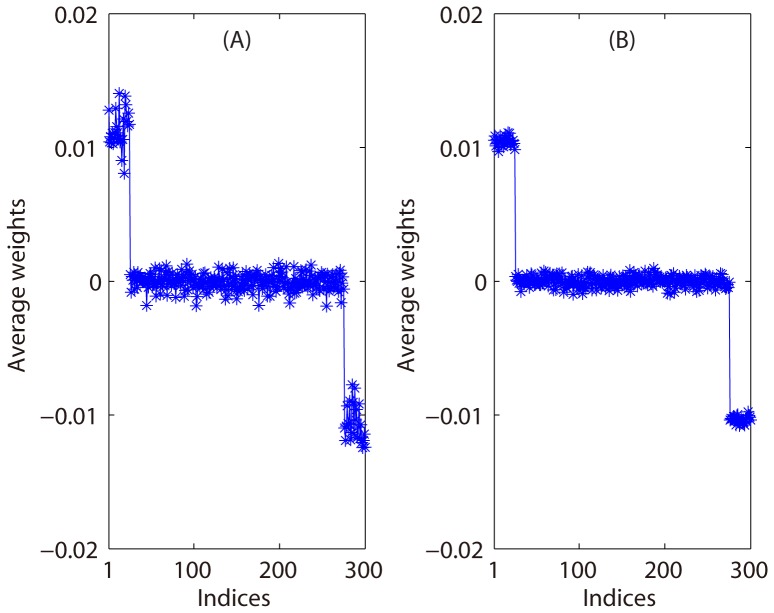
Results for the first simulation in Experiment 1. A: The average of 500 weight vectors obtained by sparse representation. B: The average of 500 weight vectors obtained by SVM.

First, we present the results of the first simulation based on equation (3). We solved the optimization problem in equation (3) for 500 times and obtained 500 weight vectors with signs. For each time, only the noise vectors 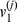 and 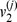, 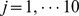 were regenerated from a Gaussian distribution with zero mean and variance 1. Using the 500 weight vectors with signs, we calculated an average weight vector, as shown in Figure 3 (A). Figure 3 (A) indicates that the patterns 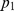 and 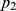 can be localized using the average weight vector, and that the signs of the average weights are associated with the two patterns. Note that the first (last) 25 entries of 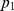 (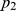) were 1 and the other entries were 0. We also used a linear SVM instead of sparse representation method to obtain another average weight vector as shown in Figure 3 (B), and the result was similar to Figure 3 (A).

**Figure 4:**
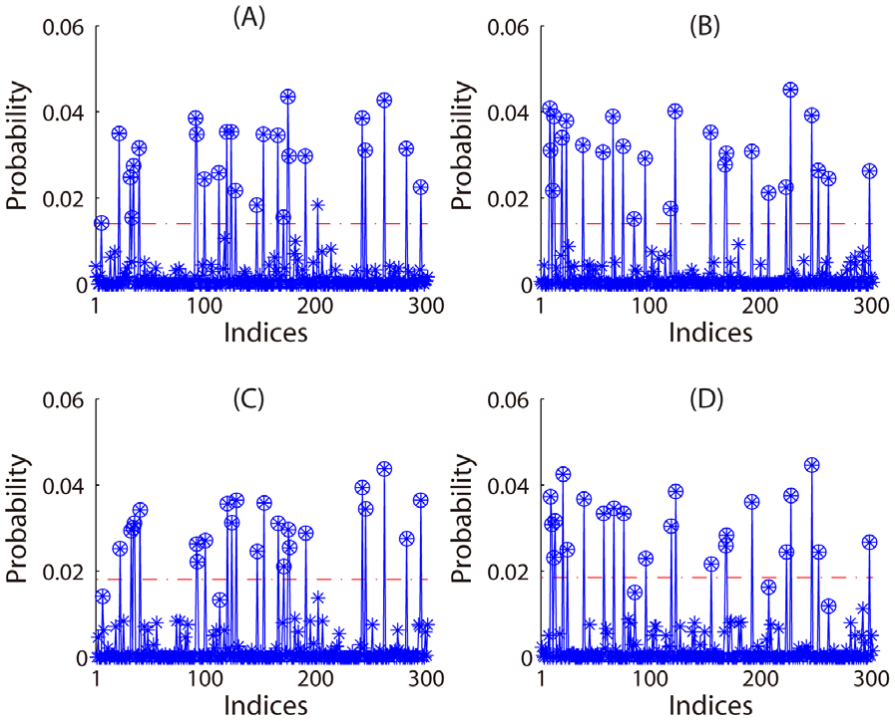
Results of our SPL algorithm with a permutation test at the group level in the second simulation of Experiment 1. A and B: with sparse representation-based weight determination; C and D: with SVM-based weight determination. Left: for the first pattern. Right: for the second pattern. In each subplot, there are an average probability density function with stars (circled or non-circled) indicating probability values, and a horizontal dash-dotted red line representing a threshold (significance level: 0.001). Stars higher than the threshold correspond to the indices of nonzeros of a predicted pattern. Those stars with circles represent the indices of the nonzeros of the true pattern.

In the following, we presented the results of the second simulation. We regenerated the data for 5 times (corresponding to 5 subjects in a real world experiment) and performed our SPL algorithm with a permutation test at the group level (see **Methods and Materials**). Using the data regenerated in each time, we obtained two probability density functions. Two average probability density functions across all the 5 times, which corresponded to the two patterns respectively, are shown in Figure 4 (A) and (B). Based on the permutation test of 100 permutations at the group level, we found two thresholds with a significance level of 0.001. For each of the two average probability density functions, we selected those variables with probability values larger than its corresponding threshold and obtained a predicted pattern. The prediction accuracy rates were 99.7% and 100% for the two patterns 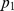 and 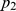 respectively. For each pattern, the prediction accuracy rate was calculated as (1-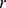)100%, where 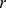 was the ratio of wrongly selected features among all the 300 features. The effectiveness of our SPL algorithm was thus demonstrated. Figure 4 (A) and (B) show the result of prediction.

We also used a linear SVM instead of sparse representation for determining the weights in our SPL algorithm and repeated the above procedure. The results are shown in Figure 4 (C) and (D). The prediction accuracy rates were 97.7% and 98.7% for the two patterns 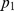 and 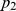 respectively. Therefore, the performance of sparse representation was comparable to that of SVM for weight determination in our SPL algorithm.

Furthermore, we considered the classification performance based on the selected informative variables. Specifically, the above 100 data vectors with labels were used as training data. Then, an independent test set in which 50 vectors contained the pattern 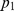 and the other 50 vectors contained the pattern 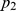 was generated. For each data vector in the training data set and the test data set, we kept those entries corresponding to all of the selected informative variables and removed the others to construct a feature vector. Using the feature vectors with labels in the training data set, we trained an SVM classifier and then predicted the labels of the test data. The classification accuracies were 82% and 75% for the sparse representation-based SPL algorithm and SVM-based SPL algorithm respectively.

Note that we localized the informative features with a high accuracy but the classification accuracy based on these informative features was relatively lower. This difference may be explained as below. First, feature selection based on the training data with labels plays an important role in classification. In order to achieve a high classification accuracy, only these features that contain strong discrimination information between the two categories are selected. In other words, not all of the informative features are selected for classification in general. However, in many real applications, e.g. neuroimaging and novelty detection/fault diagnosis, it is important to find all informative features, no matter how weak they are. We thus proposed the SPL algorithm to localize all the informative features in this study. Here, it was not an optimal way to use all of the localized informative features for classification and thus the accuracies might be lower. Second, the noise level was quite high in the simulated data sets. If the SNR was sufficiently large, we could still obtain a high classification accuracy using all of the localized informative features as shown below.

Here the parameter 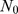 (the number of features selected in each iteration) in our SPL algorithm was set as 2. To check the robustness of our algorithm to this parameter, we set 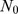 as 4 and repeated the above process. The prediction accuracy rates were 99.7% and 100% for the two patterns 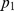 and 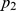 respectively. When 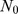 was set as 4, the average number of iterations was 6 in each fold of cross-validation, and totally there were about 600 iterations (5 subjects times 20 folds times 6 iterations). It took about 39 seconds to perform these iterations using the personal computer with dual Intel Core i5 CPU (2.67 GHz). Furthermore, it took about 3765 seconds to run our SPL algorithm with 100 permutations at the group level. We also checked the other settings for this parameter (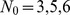) and obtained similar results. Thus our algorithm is robust to different settings of the parameter 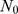.

**Figure 5:**
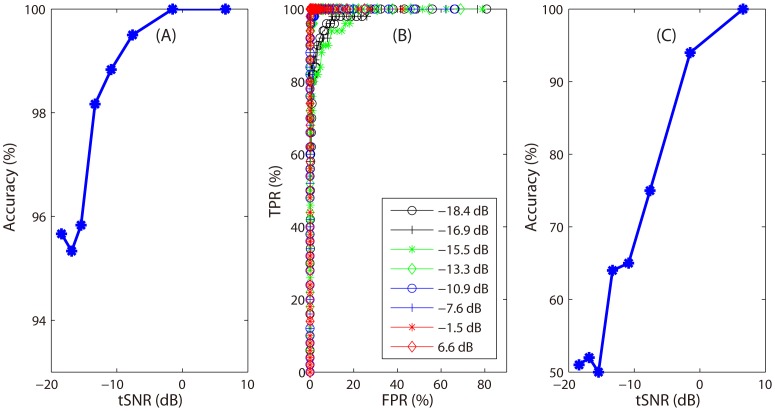
Results of our SPL algorithm at different noise levels. A: accuracy curve for localizing informative features obtained by the sparse representation-based SPL algorithm with different noise levels. B: 8 ROC curves corresponding to 8 noise levels respectively. C: accuracy curve for predicting the labels of the independent test sets generated at different noise levels.

To evaluate the robustness of our SPL algorithm to different noise level, we generated eight data sets at different tSNR values. We applied our algorithm to these data sets and obtained accuracies for localizing the two patterns and calculated ROC curves. For each noise level, we also generated an independent test set in which 50 vectors contained the pattern 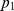 and the other 50 vectors contained the pattern 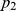, and predicted the labels of the independent test data based on the selected informative variables. Figure 5 shows the accuracy curve for localizing the informative variables (A), 8 ROC curves (B), and classification accuracy curves for the independent test data with different tSNR values (C). From Figure 5 (A), we can see that when tSNR>−10 dB, we could obtain satisfactory accuracy for localizing the two patterns.

**Figure 6:**
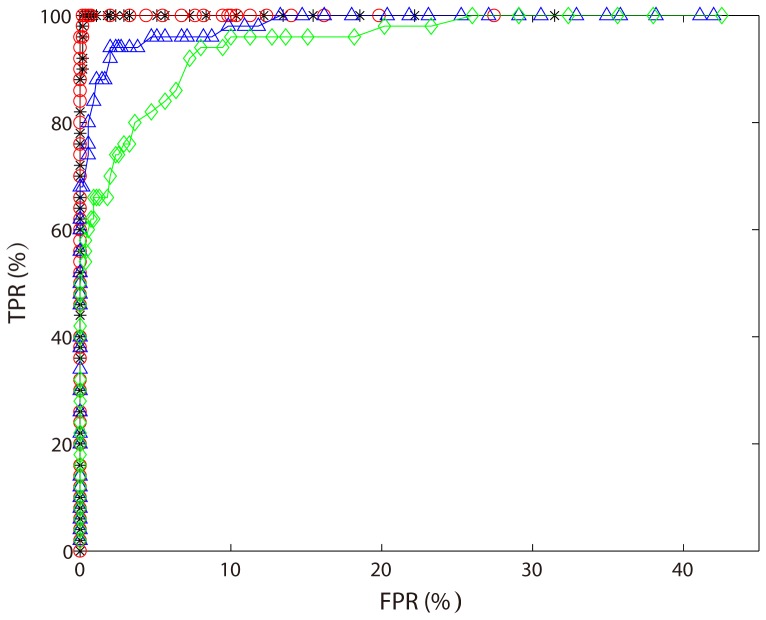
Four ROC curves obtained by our sparse representation-based SPL algorithm (black curve with stars), SVM-based SPL algorithm (red curve with circles), SVM method (blue curve with triangles) and correlation method (green curve with diamonds).

For comparison, we applied a SVM to the above data set containing 100 data vectors and obtained a 300 dimensional weight vector. For each variable, we also calculated the correlation coefficient between the 100 dimensional data vector corresponding to this variable and the label vector. We could localize the two patterns 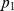 and 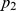 using the weight vector or these correlation coefficients. Figure 6 shows 4 ROC curves obtained by our sparse representation-based SPL algorithm (black curve with stars), SVM-based SPL algorithm (red curve with circles), SVM method (blue curve with triangles) and correlation method (green curve with diamonds). From Figure 6, we can see that sparse representation-based SPL algorithm and SVM-based SPL algorithm have comparable performance in localizing the informative patterns, which are better than SVM method and correlation method.

### Experiment 2: Optical imaging data analysis

Cells in animal early visual areas are sensitive to edge orientations and form repeated representations known as hypercolumns [22], [23]. Using optical imaging, these fine structures have been revealed in both the cat and monkey primary visual cortex [24], [25]. Here, we applied our SPL algorithm on an optical imaging data set that was collected from a Macaque monkey and determined if these columnar structures could be shown by our analysis method. We used 40 trials of horizontal and 40 trials of vertical axis-of-motion. We used a leave-one-out method, whereby there were a total of 80 folds of cross-validation. In each fold of cross-validation, we performed a recursive feature search, and 200 pixels were obtained in each iteration (100 pixels for horizontal and 100 pixels for vertical axis-of-motion).

**Figure 7:**
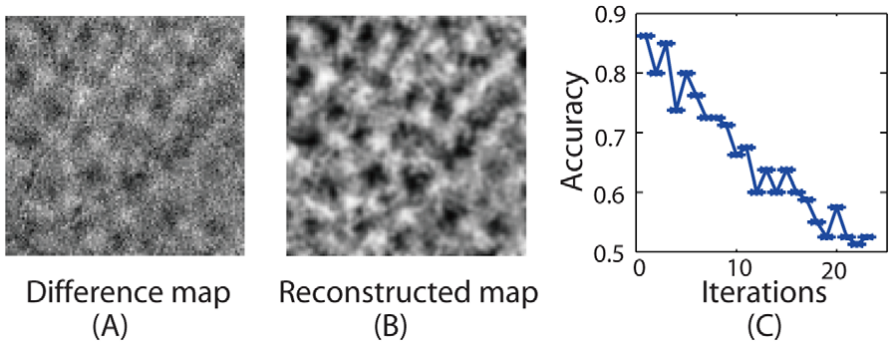
The three subplots are shown for the two classes, i.e., horizontal axis-of-motion stimuli and vertical axis-of-motion stimuli. A: a difference map between the two stimulus conditions. B: the reconstructed condition difference map between the two stimulus conditions using our approach. C: the iterative curve of decoding accuracy rates in Experiment 2.

After we finished all 80 folds of cross-validation, we counted the frequency of a pixel selected across all 80 folds and obtained two probability maps for each of the two sets of selected pixels respectively. By projecting back these pixels with probability values on a 2-dimensional map, we then calculated the difference between the two probability maps (horizontal probability map minus vertical probability map) and smoothed the difference map with a 2-D Gaussian mask (zero mean, unit variances 1 and zero covariances). The difference between the two probability maps, reflecting the class information, is shown in Figure 7 (B). For comparison, as shown in Figure 7 (A), we also obtained a differential map between these two conditions using an established method in optical imaging data analysis (the so-called differential mapping method), which allowed us to perform a subtraction of the mean maps between these two conditions.

Figure 7 shows that the dark and bright blobs in Figure 7 (B) matched those in Figure 7 (A). This demonstrates the effectiveness of our approach in searching for two classes of patterns. Furthermore, Figure 7 (C) shows that the decoding accuracy rates decrease with the iterations in each fold of cross-validation.

### Experiment 3: fMRI data analysis

Human faces share many common features. The overlap of features makes it difficult to discriminate between different semantic categories related to faces (e.g., old and young, male and female) using fMRI signals [26]. And these semantic categories were hard to be detected using the standard univariate GLM analysis method [3], [16], [17], [18]. Here, we applied our algorithm to an fMRI data set to distinguish subcategories of faces (i.e., old and young people) and to find two class-related brain patterns. We applied the GLM-SPM method to this fMRI data set and found no significant difference between the two conditions corresponding to the two classes of faces respectively. Nine subjects attended this experiment. The visual stimuli of this experiment were 80 grayscale pictures of Chinese faces at two different age levels (40 old and 40 young people). During the experiment, the subjects were instructed to make a covert semantic categorization (old vs. young) based on the presented face pictures.

For each subject, we used a leave-one-trial-out method to carry out 80 folds of cross-validation. In each fold of cross-validation, we performed a recursive feature search, and 50 voxels were obtained in each iteration (25 voxels for “old people” and the other 25 voxels for “young people”). We then obtained two probability-maps corresponding to the two age categories respectively.

**Figure 8:**
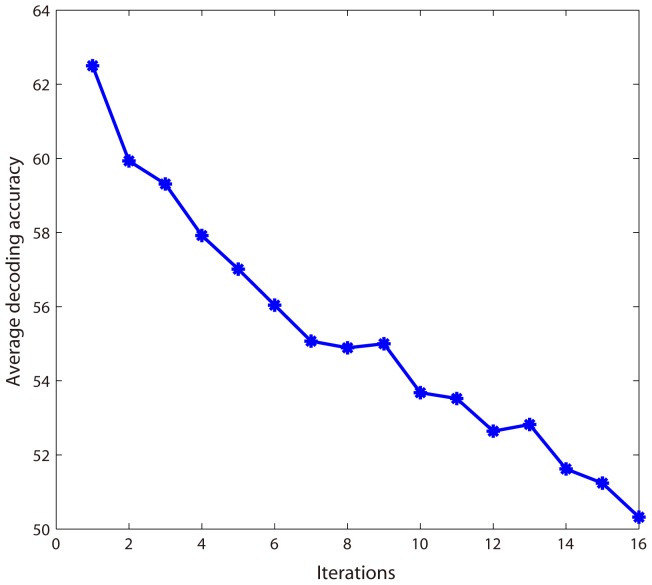
Iterative curve of average decoding accuracy rates across all the 9 subjects in Experiment 3.

Figure 8 depicts the average decoding accuracy curve across all the 9 subjects. The first accuracy was obtained using SVM classifier based on all of the 2,500 voxels initially selected by correlation coefficients, and the 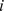-th accuracy (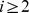) was obtained with the data updated in the 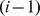-th iteration (i.e., the top 25 voxels with the highest positive weights and the top 25 voxels with the smallest negative weights were removed in the 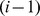-th iteration).

**Figure 9:**
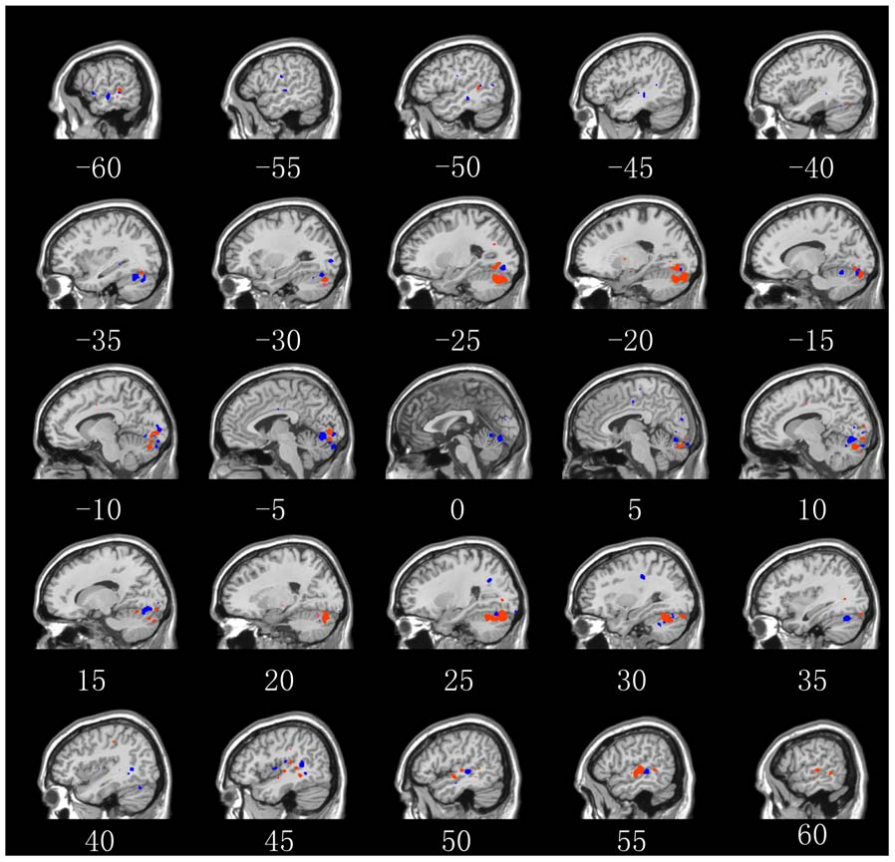
Voxels selected by our method with a significance level of 0.05 (corrected with cluster size 10) in Experiment 3. The red clusters corresponded to the “old people” stimulus condition, and the blue clusters corresponded to the “young people” stimulus condition.

For each subject, we obtained two probability maps with respect to the voxels. Two average probability maps across all the 9 subjects were then calculated. Furthermore, we performed a permutation test of 100 permutations at the group level (see **Methods and Materials**) and determined two thresholds with the significance level of 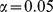. For each average probability map, we selected those voxels with probability values higher than its corresponding threshold and cluster sizes larger than 10 for multiple comparison correction, and thus obtained two sets of features corresponding to the “old people” (1506 voxels) and “young people” (1011 voxels) stimulus conditions, respectively. The clusters formed by the two sets of voxels and their corresponding brain areas are illustrated in Figure 9 and in Appendix S2.

Facial appearance changes with age [27], [28]. For example, there are changes in shape, which mainly occur through growth or weight gain or loss, and changes in the surface texture and coloration of skin and hair. Human vision appears to be sensitive to these subtle differences when determining the age of a face [29]. However, it is still not clear how these subtle differences and the related age information of faces are processed in the brain. In this study, we found distributed extrastriate areas, and most importantly, both the fusiform gyrus and the superior temporal sulcus, which are known as part of core system in face perception, were involved in the age information processing. These results clearly demonstrate that our approach can be applied to localize brain discriminative patterns related to fine perceptual differences between stimulus conditions.

## Discussion

In neuroimaging studies, the number of variables/features ranges from tens to hundreds of thousands. In comparison, only a small amount of these features are engaged in certain stimulus conditions or brain states. Furthermore, there are a limited number of trials (examples) for each stimulus condition and it is known that too many variables may lead to overfitting in pattern classification [30]. Therefore, feature selection is a necessary and challenging process when applying MVPA approaches. The feature selection procedure first aims to improve classification accuracy. Once it has been established that class information is present in a dataset, the next step is to determine where in the brain the discriminating information resides, which is known as the pattern localization process and is useful for understanding neural information coding [3], [11], [31].

In this study, we proposed a sparse representation-based method for searching for informative features. Sparse representation is a promising method for feature selection and has recently received lots of attention [31], [32], [33], [34]. Sparse representation-based feature selection can minimize overfitting in classification by automatically removing irrelevant voxels [31], since the weights of feature candidates in sparse representation are sparse (i.e. most of the weights are zero).

To improve the reliability for feature selection, we implemented a cross-validation procedure as suggested by several earlier studies [1], [31], [35]. It has been shown that the overlap between the set of voxels selected in all folds versus the set selected in an individual fold is fairly small, between 1/10 and 1/3 is typical for many fMRI datasets [36]. To improve the overlap between the features obtained in different folds, we introduced a recursive iterative elimination method [35], [37] for feature selection in each fold. In contrast to the recursive feature elimination method in the forward direction [1], [35], we used a backward elimination strategy because our main interest was to find all of the informative features existing in the dataset. Specifically, in each iteration, we first performed sparse representation and then removed the portion of the features with the highest absolute weight values. The remaining features entered into the next iteration. This procedure ran until the decoding accuracy of each iteration dropped to a threshold close to the chance level. We believe that the backward iterative elimination method provides a reasonable solution for selecting all of the informative features in our sparse based algorithm because of the following reason. In each iteration, the features with the highest absolute weight values are supposed to be informative. However, among those features with low weights, informative features may still exist. This is mainly because in a sparse representation, only a small number of informative features are used. While a feature is given a high weight, its correlated features tend to be given low weights. During the procedure of iterative elimination, the features with highest weights in an iteration are removed, those remaining informative features may be highlighted and extracted in the next iteration.

Neuroimaging data contain a large number of uninformative/noisy features that carry no useful information about the stimulus conditions. These uninformative features may be selected during each fold of the cross-validation. For instance, after the strong informative features are removed from the data set after several early iterations, weak informative features, as well as irrelevant features/noise, can be picked up under lower decoding accuracy conditions. Statistical tests are required to remove these uninformative features. Suppose that all of the informative features can be extracted in each fold, informative features that truly contribute to the discrimination should then frequently be picked up during the cross-validation. Therefore, the contribution of an informative feature can be measured as the frequency of its appearance across all folds. To remove the irrelevant features, we used a nonparametric test at the individual subject level in this study. The same idea has been used previously to generate discrimination maps [4], [14], [38]. With the assumption that the brain patterns should be the same between different subjects, we also applied another nonparametric permutation test across subjects to further remove irrelevant features. This nonparametric permutation test was similar to other group analysis methods developed for the MVPA approach [4], [14] because all of them are based on the random effects analysis [39].

The effectiveness of the above approach for selecting high percentage of the informative features as well as removing the irrelevant features was clearly demonstrated in our data analysis in Examples 1 and 2. For instance, in the optical imaging data analysis, it is known that vertical and horizontal orientations are represented as hypercolumns in the monkey primary visual cortex [24], [25], and that nearby pixels have similar orientation preferences. In addition, these fine structures (∼1 millimeter) are repeatedly represented across the cortex. As shown in Figure 7, these highly correlated features that are represented on the cortex (i.e., patches) could be extracted using our algorithm. Note this grouping effect was achieved by the iterative selection and removal in our procedure, which differed from other methods by setting a certain regularization on the classifier [33].

Besides locating these informative features in the brain, we further specified the correspondence between the groups of informative features and stimulus conditions. For instance, the ventral and dorsal visual pathway in the brain can be selectively activated by face and spatial information stimuli respectively [14], [40], [41], [42], [43]. Several studies have attempted to specify the brain activation patterns for certain stimulus conditions [2], [14], [31], [44]. Generally, a weight vector is determined to be orthogonal to the direction along which the training examples of both classes differ most. Given two classes, task 1 and task 2, with the labels +1 and −1, respectively, a positive weight means that these features/voxels present higher activity during task 1 than during task 2, and a negative weight means lower activity during task 1 than during task 2 [2], [14]. The absolute magnitude of each entry of the weight vector determines the importance of its corresponding feature in discriminating the brain states. These positive/negative weights can be thresholded so that the most important features for discriminating between cognitive states are selected. The threshold can be determined using nonparametric statistical tests, such as permutation tests. One common assumption in these studies is that the signs of weights (at least for those with large absolute values) are associated with two classes of stimuli/brain states. In this study, we also used the assumption that the sign of a weight gives class information. In the two simulations, we could estimate the two patterns (representing two classes of data) with high accuracy. In addition, in the optical imaging data analysis, we could see that the two sets of informative pixels (dark blobs and bright blobs) obtained by our algorithm were consistent with those obtained by the classic difference method. Furthermore, in Appendix S1, we presented theoretical proof for several simplified models. However, for more complex sparse representation models with high dimensionality, strictly proving the assumption remains an open problem.

## Conclusions

The multivariate pattern analysis approach is sensitive to subtle differences caused by different stimulus conditions/mental states and has been used to decode stimulus/task-related information in functional brain imaging data. In this paper, we proposed a sparse representation-based MVPA algorithm combined with a permutation test at the individual subject level or group level for feature selection in brain imaging data analysis. Two informative patterns, which correspond to two experimental conditions/brain states, respectively, were obtained. Through a recursive feature elimination procedure, most of the informative features were selected. Using the two nonparametric permutation tests, irrelevant features were significantly reduced. Applications of our methods on three experiments were presented. Specifically, data analysis results based on the toy data set and the optical imaging data set demonstrated the effectiveness of our approach, while the analysis results for an fMRI data set illustrated its application. Future work may include the extension of our algorithm to multiclass problems as well as applications in data analysis from other neuroimaging studies (e.g. neuroimaging data, medical image data).

## Supporting Information

Appendix S1 Effectiveness analysis for our sparse representation-based feature selection method.
(DOCX)

Appendix S2 Brain areas for two sets of selected voxels in Example 3.
(DOCX)

Table S1 The brain areas, volume sizes and the center coordinates of the clusters corresponding to the “old people” stimulus condition in Experiment 3. In a single brain area, at most two clusters (corresponding to the left and the right hemispheres respectively) are presented here.
(DOC)

Table S2 The brain areas, volume sizes and the center coordinates of the clusters corresponding to the “young people” stimulus condition in Experiment 3. In a single brain area, at most two clusters (corresponding to the left and the right hemispheres respectively) are presented here.
(DOC)
