# Supplementary material for: A Sparse Representation-Based Algorithm for Pattern Localization in Brain Imaging Data Analysis
Source: PLoS One. 2012 Dec 5;7(12):e50332. doi: 10.1371/journal.pone.0050332 (PMC3515601; doi:10.1371/journal.pone.0050332)
Supplement: Appendix S1 — Effectiveness analysis for our sparse representation-based feature selection method. (DOCX) [file pone.0050332.s001.docx]

**Appendix S1: Effectiveness analysis for our sparse representation-based feature selection method**

In this appendix, we present a theoretical analysis based on several simplified models to illustrate that two classes of patterns can be obtained with our sparse representation-based feature selection method. We first consider the following two models:

$\min||w||_{1},$ s.t. $(p_{1}+v_{1})w=1,$ (A1)

$\min||w||_{1},$ s.t. $(p_{2}+v_{2})w=-1,$ (A2)

where $p_{1}$ and $p_{2}$ are two $n$-dimensional row vectors representing two patterns. Without a loss of generality, suppose that the first $l_{1}$ entries of $p_{1}$ take a value of 1 and the other entries take a value of zero, and that the last $l_{2}$ entries of $p_{2}$ take a value of 1 and the other entries take a value of zero. $v_{1}$ and $v_{2}$ are two $n$-dimensional row vectors representing noise. $w$ is an $n$-dimensional weight column vector to be obtained. The optimization problems (A1) and (A2) can be transformed into two standard linear programming problems respectively [1,2].

Suppose that the *i*-th entry $p_{1i}+v_{1i}$ of the vector $p_{1}+v_{1}$ has the largest absolute value. From linear programming theory, the optimal solution of (A1) is $w_{i}=\frac{1}{p_{1i}+v_{1i}},$and $w_{j}=0$ for $j\neq i$. Note that $p_{1j}+v_{1j}=1+v_{1j}$ for $j=1,\cdots,l_{1},$ and $p_{1j}+v_{1j}=v_{1j}$ for $j=l_{1}+1,\cdots,n.$ We can prove that if the noise has a small variance, the probability of the index $i\in\{1,\cdots,l_{1}\}$ is much larger than the probability of the index $i\in\{l_{1}+1,\cdots,n\}$. Furthermore, $w_{i}=\frac{1}{p_{1i}+v_{1i}}>0$ when $i\in\{1,\cdots,l_{1}\}$. Thus we find the index of a nonzero entry of the pattern $p_{1}$ with a high probability by solving the optimization problem (A1). Furthermore, if a new noise vector (different from $v_{1}$) in (A1) is used, then similar to above, we can also find the index of a nonzero of the pattern $p_{1}$ with a high probability. This index may be different from the previous index $i\in\{1,\cdots,l_{1}\}$. In this way, we can obtain the pattern $p_{1}$ by solving the optimization problem (A1) many times (the noise vector is different each time).

Similarly, we can find the index of a nonzero of the pattern $p_{2}$ with a high probability by solving the optimization problem (A2), and obtain the pattern $p_{2}$ by solving the optimization problem (A2) many times (the noise vector is different each time). Furthermore, for the optimization problem (A2), the nonzero entry is $w_{i}=\frac{1}{p_{1i}+v_{1i}}<0$ when $i\in\{n-l_{2}+1,\cdots,n\}$.

Now we consider the following model with two constraint equations,

$\begin{matrix} \min||w||_{1},s.t. \\ \left\{\begin{matrix} (p_{1}+v_{1})w=1, \\ (p_{2}+v_{2})w=-1. \end{matrix}\right. \end{matrix}$ (A3)

According to linear programming theory, the optimal solution $w$ of (A3) has only two nonzero entries denoted as $w_{i}$ and $w_{j}$ ($i<j$), which are the solution of the following equations:

$\left\{\begin{matrix} (p_{1i}+v_{1i})w{}_{i}+(p_{1j}+v_{1j})w{}_{j}=1, \\ (p_{2i}+v_{2i})w{}_{i}+(p_{2j}+v_{2j})w{}_{j}=-1. \end{matrix}\right.$ (A4)

The solution of (A4) can be written as

$w_{i}=\frac{(p_{1j}+v_{1j})+(p_{2j}+v_{2j})}{(p_{1i}+v_{1i})(p_{2j}+v_{2j})-(p_{1j}+v_{1j})(p_{2i}+v_{2i})},$ (A5)

$w_{j}=-\frac{(p_{1i}+v_{1i})+(p_{2i}+v_{2i})}{(p_{1i}+v_{1i})(p_{2j}+v_{2j})-(p_{1j}+v_{1j})(p_{2i}+v_{2i})},$ (A6)

where the absolute values of $w_{i}$ and $w_{j}$ should be as small as possible because they are the non-zeros of the solution of the optimization problem (A3).

For simplicity, we suppose that the noise is from a distribution (e.g. Gaussian distribution) with zero mean and a small variance. Here we use the assumption that the noise variance is small for the convenience of mathematical deduction. In fact, in a real world application, this strict assumption is not necessary (see Example 1 in this paper). Then with high probability, the absolute value of each entry of noise vectors is much smaller than 1, and $(p_{kl}+v_{kl})>0$, for $k=1,2$ and $l=i,j$. We first consider an ideal case.

1) Ideal case: $i,j$ belong to the index sets $\{1,\cdots,l_{1}\}$and $\{n-l_{2}+1,\cdots,n\}$respectively (note: because $i<j$, $i,j$ cannot belong to the index sets $\{n-l_{2}+1,\cdots,n\}$and $\{1,\cdots,l_{1}\}$, respectively). In this case, (A5) and (A6) become

$w_{i}=\frac{p_{2j}+v_{1j}+v_{2j}}{(p_{1i}+v_{1i})(p_{2j}+v_{2j})-v_{1j}v_{2i}},$ (A7)

$w_{j}=-\frac{p_{1i}+v_{1i}+v_{2i}}{(p_{1i}+v_{1i})(p_{2j}+v_{2j})-v_{1j}v_{2i}}.$ (A8)

Suppose that the noise $v_{ki},v_{kj}$ ($k=1,2$) are sufficiently small with a high probability. Then $w_{i}$and $w_{j}$ are close to 1 and -1 respectively with high probability. Thus in this case, we find the index of a nonzero of pattern $p_{1}$ corresponding to a positive entry of $w$, and the index of a nonzero of pattern $p_{2}$ corresponding to a negative entry of $w$ by solving (A3).

Next, we consider the other possible cases and prove that these cases can be excluded.

2) $w_{i}$ and $w_{j}$cannot be simultaneously larger than zero (smaller than zero). Otherwise, the two equalities in (A4) do not simultaneously hold since all of the coefficients are positive.

3) The indices $i,j$ cannot belong to one of the three index sets $\{1,\cdots,l_{1}\}$, $\{l_{1}+1,\cdots,n-l_{2}\}$, and $\{n-l_{2}+1,\cdots,n\}$ simultaneously. Otherwise, $w_{i}$ and $w_{j}$ have larger absolute values than those obtained in the above ideal case. For instance, if $i,j$ simultaneously belong to the index set $\{1,\cdots,l_{1}\}$, then $w_{i}$ and $w_{j}$in (A5) and (A6) become

$w_{i}=\frac{p_{1j}+v_{1j}+v_{2j}}{(p_{1i}+v_{1i})v_{2j}-(p_{1j}+v_{1j})v_{2i}},$ (A9)

$w_{j}=-\frac{p_{1i}+v_{1i}+v_{2i}}{(p_{1i}+v_{1i})v_{2j}-(p_{1j}+v_{1j})v_{2i}}.$ (A10)

Because the noise is small, the common dominator in (A9) and (A10) is small. Thus, the absolute values of $w_{i}$ and $w_{j}$ are generally much larger than 1.

4) The indices $i,j$ cannot belong to the two index sets $\{1,\cdots,l_{1}\}$ and $\{l_{1}+1,\cdots,n-l_{2}\}$ or two index sets $\{l_{1}+1,\cdots,n-l_{2}\}$ and $\{n-l_{2}+1,\cdots,n\}$, respectively. This can be proven in a manner similar to that used above.

It follows from the above analysis that the above ideal case 1) happens with a high probability when the noise is small. Furthermore, using different noise vectors, we can solve (A3) many times to obtain the patterns $p_{1}$ and $p_{2}$. This has been demonstrated via simulation (data not shown). Note that there are only two constraint equations for (A3). For more constraint equations, the situation is much more complex and it is difficult for us to analyze it as above. However, the conclusion is still true, which was demonstrated in the first simulation in Experiment 1.
