# Supplementary material for: A Sparse Representation-Based Algorithm for Pattern Localization in Brain Imaging Data Analysis
Source: PLoS One. 2012 Dec 5;7(12):e50332. doi: 10.1371/journal.pone.0050332 (PMC3515601; doi:10.1371/journal.pone.0050332)
Supplement: Appendix S2 — Brain areas for two sets of selected voxels in Example 3. (DOCX) [file pone.0050332.s002.docx]

**Appendix S2: Brain areas for two sets of selected voxels in Example 3**

The following two tables (Tables S1 and S2) present the brain areas for the two sets of selected voxels in Example 3.
