# Supplementary material for: A Sparse Representation-Based Algorithm for Pattern Localization in Brain Imaging Data Analysis
Source: PLoS One. 2012 Dec 5;7(12):e50332. doi: 10.1371/journal.pone.0050332 (PMC3515601; doi:10.1371/journal.pone.0050332)
Supplement: Table S1 — The brain areas, volume sizes and the center coordinates of the clusters corresponding to the “old people” stimulus condition in Experiment 3. In a single brain area, at most two clusters (corresponding to the left and the right hemispheres respectively) are presented here. (DOC) [file pone.0050332.s003.doc]

| Brain Region | BA | Lat. | Volume (mm^3^) | Talairach Coordinates | | |
| --- | --- | --- | --- | --- | --- | --- |
|  |  |  |  | x | y | z |
| Lingual Gyrus | 17/18 | L | 786 | -6 | -84 | 3 |
| Middle Temporal Gyrus | 21/22/37 | L | 231 | -57 | -38 | 6 |
|  |  | R | 310 | 57 | -47 | 2 |
| Fusiform Gyrus | 19 | L | 660 | -24 | -68 | -6 |
|  |  | L | 179 | -39 | -69 | -11 |
| Middle Occipital Gyrus | 18 | R | 84 | 28 | -80 | 2 |
| Superior Temporal Gyrus | 21/22/39 | R | 1359 | 54 | -28 | 5 |
|  |  | L | 183 | -49 | -46 | 8 |
| Cuneus | 17/18 | R | 105 | 8 | -90 | 8 |
| Inferior Occipital Gyrus | 17 | R | 329 | 13 | -89 | -6 |
| Precuneus | 7 | L | 35 | -26 | -70 | 24 |
| Inferior Parietal Lobule | 40 | R | 58 | 38 | -30 | 42 |
| Cingulate Gyrus | 24 | L | 26 | -10 | -3 | 36 |
|  |  | R | 26 | 10 | -7 | 37 |
| Insula | 13 | L | 58 | -34 | 29 | 17 |
| Sub-Gyral |  | R | 173 | 45 | -46 | -2 |
| Declive |  | R | 4506 | 30 | -60 | -18 |
|  |  | L | 5539 | -27 | -73 | -22 |
| Thalamus |  | R | 26 | 21 | -18 | 0 |
| Lentiform Nucleus |  | L | 53 | -20 | -2 | 8 |
