# Supplementary material for: A Sparse Representation-Based Algorithm for Pattern Localization in Brain Imaging Data Analysis
Source: PLoS One. 2012 Dec 5;7(12):e50332. doi: 10.1371/journal.pone.0050332 (PMC3515601; doi:10.1371/journal.pone.0050332)
Supplement: Table S2 — The brain areas, volume sizes and the center coordinates of the clusters corresponding to the “young people” stimulus condition in Experiment 3. In a single brain area, at most two clusters (corresponding to the left and the right hemispheres respectively) are presented here. (DOC) [file pone.0050332.s004.doc]

| Brain Region | BA | Lat. | Volume (mm^3^) | Talairach Coordinates | | |
| --- | --- | --- | --- | --- | --- | --- |
|  |  |  |  | x | y | z |
| Lingual Gyrus | 17/18 | L | 2138 | -4 | -75 | -3 |
|  |  | R | 876 | 11 | -74 | -7 |
| Middle Temporal Gyrus | 21/22/37 | L | 84 | -57 | -38 | 3 |
|  |  | R | 110 | 43 | -54 | 0 |
| Fusiform Gyrus | 19 | L | 143 | -19 | -81 | -10 |
|  |  | R | 11 | 42 | -66 | -8 |
| Middle Occipital Gyrus | 18 | L | 586 | -27 | -79 | -5 |
| Superior Temporal Gyrus | 21/22/39 | R | 609 | 50 | -33 | 4 |
|  |  | L | 289 | -59 | -23 | -2 |
| Cuneus | 17/18 | L | 517 | -12 | -93 | 4 |
|  |  | R | 113 | 2 | -83 | 16 |
| Inferior Occipital Gyrus | 17 | R | 86 | 23 | -98 | -10 |
|  |  | L | 115 | -12 | -90 | -8 |
| Precuneus | 7 | R | 137 | 26 | -65 | 28 |
| Cingulate Gyrus | 24 | L | 13 | -6 | -10 | 30 |
|  |  | R | 26 | 6 | -12 | 39 |
| Middle Frontal Gyrus | 6 | R | 17 | 6 | -23 | 58 |
| Postcentral Gyrus | 2 | R | 223 | 32 | -30 | 33 |
| Insula | 13 | R | 139 | 44 | -12 | 8 |
|  |  | L | 114 | -52 | -19 | 22 |
| Inferior Temporal Gyrus | 19 | L | 17 | -51 | -63 | 0 |
| Parahippocampal Gyrus | 19 | L | 26 | -38 | -53 | -1 |
| Declive |  | R | 791 | 37 | -67 | -20 |
|  |  | L | 471 | -37 | -61 | -18 |
| Caudate |  | L | 56 | -38 | -37 | 0 |
| Culmen |  | R | 104 | 29 | -49 | -26 |
